# A Novel Post-Processing Method Based on a Weighted Composite Filter for Enhancing Semantic Segmentation Results

**DOI:** 10.3390/s20195500

**Published:** 2020-09-25

**Authors:** Xin Cheng, Huashan Liu

**Affiliations:** 1 College of Information Science and Technology, Donghua University, Shanghai 201620, China; xin_cheng@mail.dhu.edu.cn; 2 Engineering Research Center of Digitized Textile and Apparel Technology, Ministry of Education, Shanghai 201620, China

**Keywords:** image semantic segmentation, post-processing enhancement, weighted composite filter (WCF), guided image filter, minimum spanning tree (MST)-based filter

## Abstract

Image semantic segmentation is one of the key problems in computer vision. Despite the enormous advances in applications, almost all the image semantic segmentation algorithms fail to achieve satisfactory segmentation results due to lack of sensitivity to details, or difficulty in evaluating the global similarity of pixels, or both. Posting-processing enhancement methods, as the outstandingly crucial means to ameliorate the above-mentioned inherent flaws of algorithms, are almost based on conditional random fields (CRFs). Inspired by CRFs, this paper proposes a novel post-processing enhancement framework with theoretical simplicity from the perspective of filtering, and a new weighted composite filter (WCF) is designed to enhance the segmentation masks in a unified framework. First, by adjusting the weight ratio, the WCF is decomposed into a local part and a global part. Secondly, a guided image filter is designed as the local filter, which can restore boundary information to present necessary details. Moreover, a minimum spanning tree (MST)-based filter is designed as the global filter to provide a natural measure of global pixel similarity for image matching. Thirdly, a unified post-processing enhancement framework, including selection and normalization, WCF and argmax, is designed. Finally, the effectiveness and superiority of the proposed method for enhancement, as well as its range of applications, are verified through experiments.

## 1. Introduction

Image semantic segmentation [1,2] refers to the pixel level segmentation and marking of different kinds of objects from the image, and it is widely applied into various fields such as aerospace, military, intelligent driving, multimedia, medicine, and so on.

A majority of popular learning methods for image semantic segmentation are mainly based on fully convolutional network (FCN) [3], which greatly improves the segmentation accuracy and is considered as the cornerstone of this research field [4]. Nowadays, researches are conducted successively to look for improved or new semantic segmentation algorithms [5,6,7,8,9,10,11]. A semi-supervised multilabel FCN for hierarchical object parsing of images is presented in [6]. A systematic way to utilize both global and local contextual information in a single network is investigated in [7]. In the meanwhile, a global-and-local network architecture (GLNet) is proposed in [9] to incorporate global spatial information and dense local multi-scale context information, so as to model the relationship between objects in a scene. To efficiently exploit context, two types of attention modules are appended on the top of the dilated FCN in [8]. Furthermore, challenges of learning spatial context for the semantic segmentation are addressed by using the Deep Convolutional Neural Networks (DCNNs) in [10] and a novel approach superpixel-enhanced deep neural forest is proposed to target the blur on object boundaries caused by DCNN-based semantic segmentation methods in [11].

However, the inherent invariance to spatial transformations of convolutional neural networks (CNN) architectures [12] makes almost every method still has the following problems [13]: (1) The methods of up-sampling are not sensitive to the details in images, even if networks would be introduced into them. Furthermore, the results of up-sampling are still fuzzy and smooth. (2) The relationship among pixels is not fully considered and the spatial regularization step used in the general pixel classification-based segmentation method is neglected, which makes segmentation networks lack spatial consistency. (3) Although deep neural networks effectively extract local features and make good predictions by using small receptive fields, the ability to model directly by global context information and predict interactions is absent.

For the problems mentioned above, which limit the application of image semantic segmentation algorithm to some extent, researchers resort to the post-processing method, conditional random fields (CRF). Combining the structured modeling capabilities of CRFs with the feature extraction power of CNNs, the segmentation results can indeed be enhanced, and more satisfactory masks can be generated [14,15,16,17,18,19,20]. A structured prediction technique involving the virtues of Gaussian conditional random fields (G-CRF) is proposed in [14]. Repeated expensive CRF inference for back propagation gets alleviated by efficient piecewise training in [15]. Besides, for heavy computation problems, only local-range CRFs are used to refine the masks of semantic image segmentation in [16], which can alleviate the problem (1) and problem (2) to some degree, but can not work on problem (3). Thus, [17] presents an efficient mean-field approximation inference method for fully-connected CRFs, which is sensitive to initialization and make strong assumptions, and then [18] proposes an efficient yet general semi-definite programming algorithm with fast computation. Subsequently, dense CRFs are used as the post-processing of deep-learning-based segmentation for their accurate boundary recovery ability [19,20]. The dense CRFs significantly reduce the computational complexity and make full use of the global context information. However, they are unfriendly to real-time applications and are hard to optimize. Moreover, the complicated theory is also not conducive to its practical application.

To the best of our knowledge, almost all the existing post-processing methods are based on CRFs, and the design of post-processing methods from the perspective of filtering has not been investigated. To meet the challenges mentioned above in a unified framework, for the first time, we propose a novel post-processing enhancement method with theoretical simplicity and effectivity from the perspective of filtering for accurate semantic segmentation and labeling. The main contributions of this paper can be summarized as follows:

- A new post-processing strategy with a unified framework is proposed. It consists of three consecutive sub-strategies, including (i) Selection and normalization, (ii) Weighted composite filter (WCF), and (iii) Argmax. It provides a new enhancement solution for semantic segmentation results outside the framework based on CRFs.
- A novel WCF is proposed, in which a local guided image filter and a minimum spanning tree (MST)-based filter are combined by adjustable weights.
- Compared with the complex theory and structure of CRFs, the proposed enhancement method can combine advantages of the local characteristics of boundary protection and the global characteristics of recognizing global similarity, and is qualified to solve the inherent problems in the semantic segmentation algorithms with theoretical simplicity.

## 2. Related Work

The proposed post-processing method combining a local edge-preserving filtering technique with MST accumulation for an image aims to improve multi-class image segmentation and enhance the masks of image semantic segmentation. Local image filters and MST for an image are the most related topics to this paper.

### 2.1. Local Image Filter

Recently, the novel image filter [5,21,22] enhancing the image in real time has become a research hotspot. The essence of image filters for image enhancement is to separate noise from pixel observations and recover the color and characteristics of the pixel itself. The most local image filters [5,23,24,25,26,27,28,29,30] are of good performance of edge-preserving and good ability to recover the details of input images, such as Gaussian filter [23], bilateral filter [24], improved bilateral filter (including adaptive bilateral filter [25,26], joint bilateral filter [29,30], and so on), and guided filter [27,28], etc, which have the potential to solve problem (1) mentioned in Section 1. Although the Gaussian filter and the (improved) bilateral filter which is the weighted nonlinear filter based on the improvement of Gaussian filter have the characteristics of easy implementation, non-iteration, and stable filtering effects, the amount of calculation is still large and may generate gradient inversion phenomena of different degrees, which is not conducive to real-time applications [24,25,29]. Compared with the above filters, the computational complexity of the guided filter as a local linear filter is independent of the size of the filtering window, which means that it may be more efficient when processing a large mass of images. Therefore, for real-time considerations, it is designed as the local filtering part of WCF.

**Figure 1:**
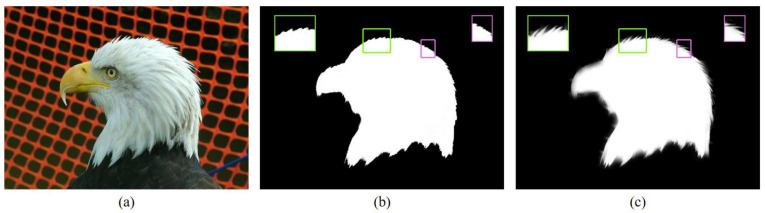
Edge-preserving filtering results of a binary image using local guided filter. (**a**) is the guide image. (**b**) is the input image. (**c**) is the guided filter result.

As shown in Figure 1, the local filter can significantly recover lost hair details and preserve the edge. Besides, local image filters are all based on windows when solving enhancement problem, which means it could improve and resolve problem (2) to some extent.

The general local linear filtering process can be defined as a weighted sum on a pixel support region centered at pixel *i*, i.e.,

(1) $$\begin{array}{c} q_{i}=\sum_{j}\omega_{ij}\left(I\right)p_{j}, \end{array}$$

where *j* is the pixel index in the filter window, and $\omega_{ij}\left(I\right)$ is the weight of guidance *I* which can be regarded as the coherence between the center pixel *i* and pixel *j* in the support window.

### 2.2. Minimum Spanning Tree (MST) for Global Filtering

Aiming at the scale defect of the local filter, identifying global characteristics and connecting global information is a potentially effective means. Referece [31] indicates that the image segmentation process can be handled as a clustering problem and the MST can preserve the connectivity of the image graph and can provide a link to all nodes at a minimum total edge cost during clustering, which is verified in follow-up researches [32,33]. An efficient MST based global filtering method for image matching is first proposed in [32]. Furthermore, the improvements have been made to address limitations for data sets with different density distribution in [33]. Besides, compared with the uncertainty of connectivity and the complexity of solving the non-deterministic polynomial (NP)-hard problem in the normalized cutting method [34,35], the MST can preserve all the important edge information without requiring any closing or connection of the edges and its pixels spatial relationship provides the possibility of fusion with local filtering algorithms. Consequently, the MST is designed for the global filtering part of WCF.

**Figure 2:**
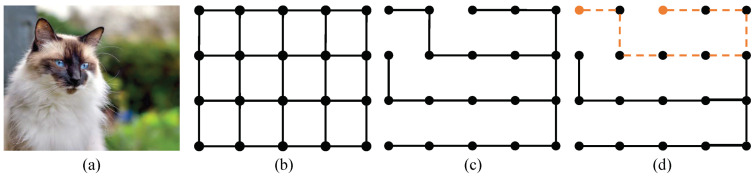
A simple illustration of the MST for an image. (**a**) is an image. (**b**) is a 4-connected, undirected graph built by (**a**). (**c**) is the MST generated from (**b**). (**d**) shows the path on MST between two target nodes.

The MST structure [33] for an image which involves pixel spatial distance and color/intensity difference provides a natural measure of global pixel similarity and has the potential to solve the problem (3). MST for an image regards an image as a 4-connected, undirected graph G=(V,E), where V is the vertex formed by all the pixels in the image and E is the edge constituted by all edges between connected pixels. A simple illustration of the minimum spanning tree for an image is shown in Figure 2.

The weight of an edge connecting two pixel nodes *u* and *v* is defined as:

(2) $$\begin{array}{c} e\left(u,v\right)=e\left(v,u\right)=|I_{u}-I_{v}|, \end{array}$$

where Kruskal or Prime method is utilized on graph *G* to obtain the MST structure.

The similarity *S* between any two nodes *i* and *j* is:

(3) $$\begin{array}{c} S\left(i,j\right)=S\left(j,i\right)=\exp\left(-\frac{L(i,j)}{\sigma }\right), \end{array}$$

where σ controls the sensitivity of the similarity between *i* and *j*, L(i,j) means the length on MST between *i* and *j* (the sum of the weights of the edges on the path from *i* to *j* in the MST).

## 3. Method

A vital contribution of the proposed method is that local image filter and MST for an Image can be applied to enhance semantic image segmentation results by effectively ameliorating three problems mentioned in the introduction part. In this part, the proposed filter and the enhancement method based on the proposed filter will be described to present the simplicity and effectivity of the theory.

### 3.1. Definition of Weighted Composite Filter (WCF)

The proposed weighted composite filter (WCF) is composed of local filtering part and global filtering part, which not only can identify local details but also has the capability of global information utilization and spatial consistency.

Reference [36] and theoretical analysis in pervious sections indicate that the information of original images can be used to enhance $P\left(x_{i}\right)=\left\{p_{L}|L=1,2,\;\cdot \;\;\cdot \;\;\cdot \;,k\right\}$, where $P(x_{i})$ denotes the label assignment probability at pixel *i* as computed by a neural network and *L* denotes labels. Following this idea, a guided image filter of local linear type, which can utilize a guidance image relevant to the input image to enhance the input image, is chosen as the local filtering part of WCF during filtering. Meanwhile, based on the hypothesis that the MST structure of the original image has the close relationship with ideal label assignment probability $Q\left(x_{i}\right)=\left\{q_{L}|L=1,2,\;\cdot \;\;\cdot \;\;\cdot \;,k\right\}$, an MST-based filter is designed as the global filtering part of MCF.

#### 3.1.1. Guided Image Filter for Local Filtering

According to the principle of guided filter [27,28], it is assumed that for each kind of label, the guided image filter is a local linear model between the original image *I* and the ideal label assignment probability $q_{L}\left(x_{i}\right)$, and the ideal label assignment probability can be considered to be the label assignment probability gained by subtracting unwanted components that may be made by the defects of neural network from a neural network. For each label, the output of guided filter $q_{L}\left(x_{i}\right)$ at a pixel *i* is

(4) $$\begin{array}{c} \;q_{L}\left(x_{i}\right)=\sum_{j}\omega_{ij}^{L}\left(I\right)p_{L}\left(x_{j}\right), \end{array}$$

(5) $$\begin{array}{c} \omega_{ij}^{L}\left(I\right)=\frac{1}{{\left|W\right|}^{2}}\sum_{(i,j)\in W}\left[1+\frac{\left(I_{i}-\mu_{W}\right)\left(I_{j}-\mu_{W}\right)}{\sigma_{W}^{2}+\varepsilon }\right], \end{array}$$

where $\omega_{ij}^{L}\left(I\right)$ is the weight between pixel *i* and *j* for label *L*, *j* represents the pixel index in the window *W*, *i* is the center of the window, *W* is a square area with a radius of *r*, $\mu_{W}$ and $\sigma_{W}$ denote the mean and variance of guidance *I* in window *W*, respectively, |W| is the pixel number in *W*. The filtering process is controlled by two parameters: the regularization parameter ε and the window radius *r*. Note that, the output of guided filter is still a label assignment probability, in which the larger value part indicates the higher probability of belonging to this label class.

#### 3.1.2. MST-Based Filter for Global Filtering

For each kind of label, the label assignment probabilities of two pixels *i* and *j* belonging to the same object are akin to the similarity *S* between nodes *i* and *j* on MST, whose structure can be obtained from the original image by (2) and (3). The MST-based global filter can be defined as

(6) $$\begin{array}{c} q_{L}\left(x_{i}\right)=\frac{\sum_{j}S_{I}\left(i,j\right)p_{L}\left(x_{j}\right)}{\sum_{j}S_{I}\left(i,j\right)}, \end{array}$$

where $S_{I}\left(i,j\right)$ is the similarity between nodes *i* and *j*, and detailed definition can be seen in (3), *j* is the pixel index in the whole image and $\sum_{j}S_{I}\left(i,j\right)$ is the normalization factor.

Combining the benefits of two filters mentioned above, the final WCF can be designed as

(7) $$\begin{array}{c} q_{L}\left(x_{i}\right)=\omega_{1}\frac{\sum_{j}S_{I}\left(i,j\right)p_{L}\left(x_{j}\right)}{\sum_{j}S_{I}\left(i,j\right)}+\omega_{2}\frac{1}{{\left|W\right|}^{2}}\sum_{(i,j)\in W}\left[1+\frac{\left(I_{i}-\mu_{W}\right)\left(I_{j}-\mu_{W}\right)}{\sigma_{W}^{2}+\varepsilon }\right] \end{array}$$

where $\omega_{1}$ and $\omega_{2}$ denote the weight of local part and global part of WCF, respectively, and $\omega_{1}+\omega_{2}=1$.

### 3.2. Enhancement Method Based on WCF

**Figure 3:**
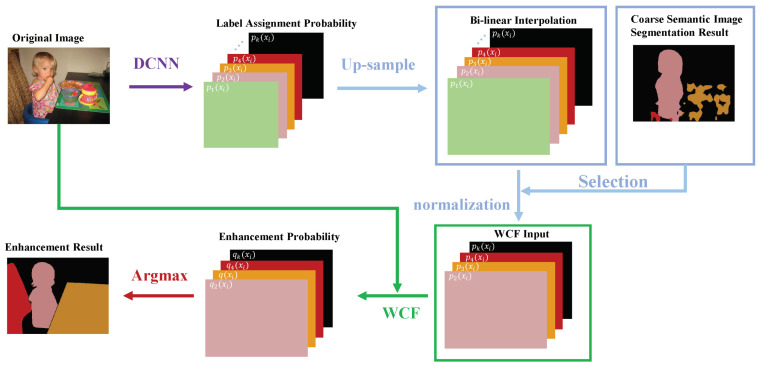
WCF-based enhancement method illustration. The size of the label assignment probability $P(x_{i})$ computed by a deep convolutional neural networks (DCNN) is different from the original image. (i) A bi-linear interpolation stage enlarges the probability maps to the original image resolution. Then, select the classes occurring in the coarse segmentation mask to do normalization. (ii) Use WCF to improve the probability maps. (iii) Use the argmax function to obtain final segmentation result.

In this section, an image semantic segmentation enhancement method based on WCF is proposed. The framework of this method can be divided into three parts: (i) Selection and normalization, (ii) WCF, and (iii) argmax, as shown in Figure 3.

(i) **Selection and normalization**. Since the size of the label assignment probability $P(x_{i})$ computed by a deep convolutional neural network (DCNN) [37] is different from the original image, an up-sampling operation ought to be used to resize the probability. For original probability, we utilize bi-linear interpolation to reach the original image resolution. For the assumption that only the classes occurring in the coarse semantic image segmentation mask would influence the segmentation results, only the assignment probability of class labels is chosen for improvement methods instead of using all classes, which reduces the computed quantity. Meanwhile, the uncertain range of values and output values of DCNN for each pixel and each class are normalized to the same order as the image pixel values. Only in this way can the label assignment probability get updated, and the filtering process would be effective. Blue arrows in Figure 3 represent the step (i).
(ii) **WCF**. For each *L* in coarse mask, $P\left(x_{i}\right)=\left\{p_{L}\left(x_{i}\right)|L\in \mathrm{caorse}\;\mathrm{mask}\right\}$ is improved by (7). Then, the Scene segmentation with dual relation-aware attention network.enhancement probability $Q\left(x_{i}\right)=\left\{q_{L}\left(x_{i}\right)|L\in \mathrm{caorse}\;\mathrm{mask}\right\}$ can be obtained. Green arrows in Figure 3 represent the step (ii).
(iii) **Argmax**. The argmax function [38] of each pixel-bit depth vector is used to decompose the predicted values into segmentation masks and to get the enhancement result. The enhancement method based on WCF is then employed to improve the segmentation result and better capture the object boundaries. Red arrows in Figure 3 represent the step (iii).

## 4. Experiments

To validate the enhancement performance, the proposed enhancement method based on WCF is evaluated by comparative experiments on the challenging PASCAL VOC 2012 image data set. In order to demonstrate the effectiveness of the method and its wide application, the proposed method is applied into four mainstream image semantic segmentation methods, i.e., FCN (which can accept input images of any size and contains the deconvolution layer that can output elaborate results and the skip structure to ensure robustness and accuracy) [4], FastFCN (which proposes the joint pyramid up-sampling module and applies the merged pyramid up-sampling module to reduce the amount of calculation and improve the accuracy) [39], DeepLabv3 (which proposes atrous spatial pyramid pooling (ASPP) to robustly segment objects at multiple scales and combines DCNN and probability model to improve the detection boundary of the object) [19] and pyramid scene parsing network (PSPNeT) (which exploits the capability of global context information by different-region-based context aggregation through pyramid pooling module together with the PSPNet) [40], to be new methods, i.e., FCN+WCF, FastFCN+WCF, DeepLab+WCF and PSPNET+WCF, and then their final segmentation effects are compared.

### 4.1. Training and Parameters Selection

The data set is divided into verification set and test set. Following the literature [41], the additional annotation with labelled images is used for training. Furthermore, then we hold 100 images from VOC 2012 for fine-tuning the paraments and verification. The parameter ε is fixed as ε=0.1×0.1. The parameters of FCN are set as: σ=25, r=15 and $\omega_{1}=0.6$. The parameters of FastFCN are set as: σ=25, r=10 and $\omega_{1}=0.9$. The parameters of DeepLab are set as: σ=30, r=5 and $\omega_{1}=0.9$. The parameters of PSPNet are set as: σ=25, r=5 and $\omega_{1}=0.6$.

In the process of validation, different target objects obtained by segmentation are marked with different colors, and objects of the same type in different pictures are marked with the same color. The four benchmark methods used for comparison, which are tuned to the optimal state that is the same accuracy standards in the respective literature to obtain the above-mentioned fixed parameters perform semantic segmentation tasks on the data set to obtain their respective processing results. Then, the four methods are combined with the proposed post-processing method to perform semantic segmentation on the data set again.

### 4.2. Evaluation

**Figure 4:**
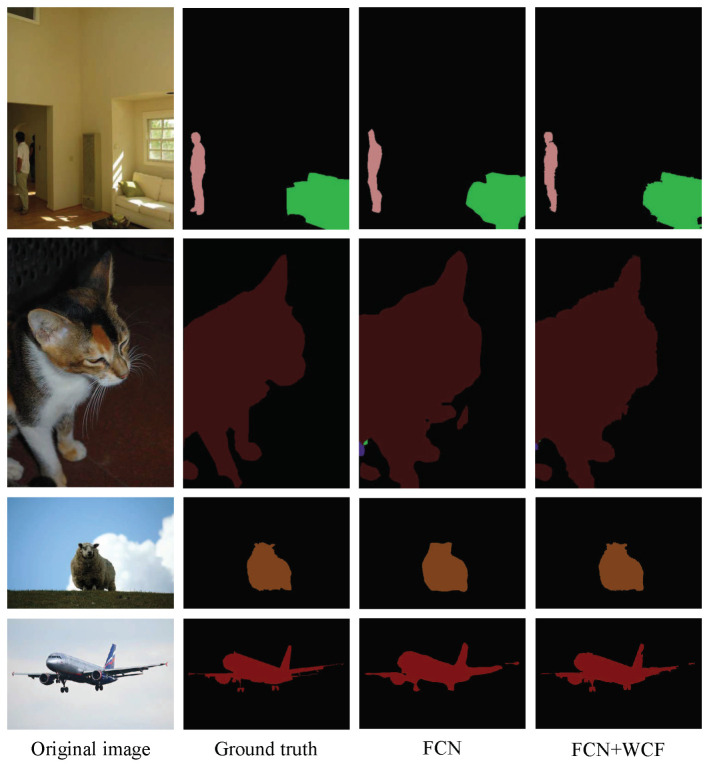
Enhancement of segmentation results of FCN. From the first row to the fourth row are man, cat, sheep and aircraft, respectively. From the first column to the fourth column are the original images, the ground truth of images, the preliminary segmentation results by FCN and enhancement results by the proposed methods FCN+WCF.

**Figure 5:**
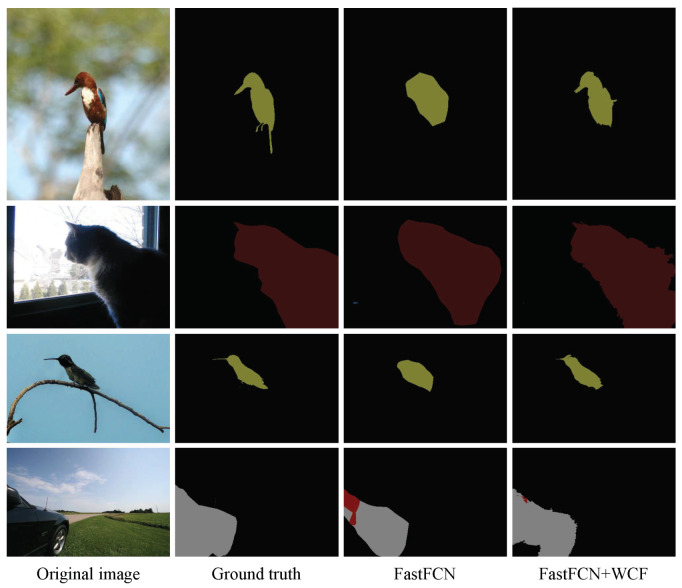
Enhancement of segmentation results of FastFCN. From the first row to the fourth row are bird, cat, bird and car, respectively. From the first column to the fourth column are the original images, the ground truth of images, the preliminary segmentation results by FastFCN and enhancement results by the proposed methods FastFCN+WCF.

**Figure 6:**
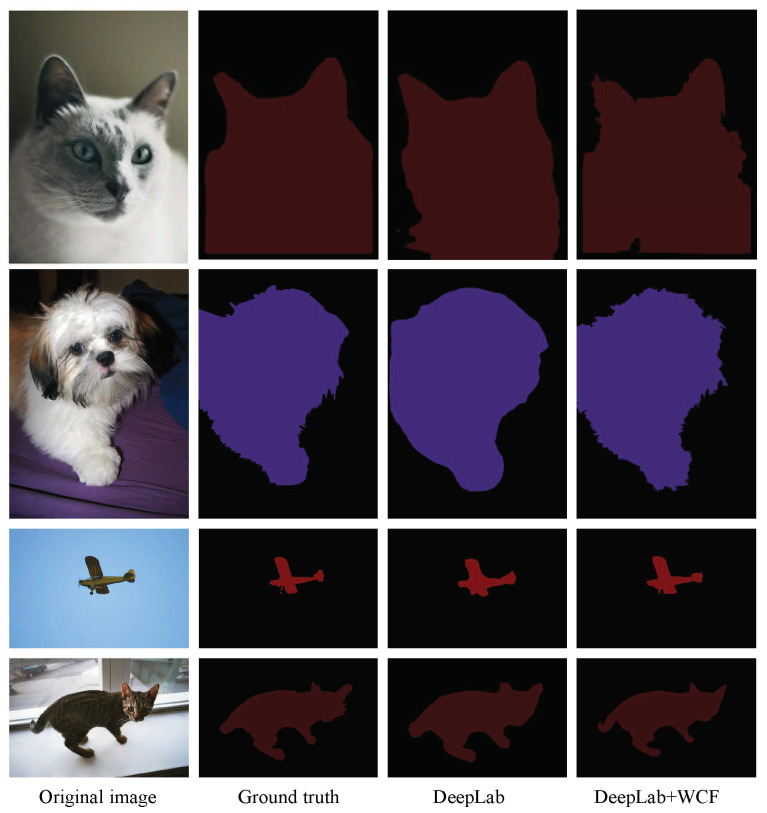
Enhancement of segmentation results of DeepLab. From the first row to the fourth row are cat, dog, aircraft and cat, respectively. From the first column to the fourth column are the original images, the ground truth of images, the preliminary segmentation results by DeepLab and enhancement results by the proposed methods DeepLab+WCF.

**Figure 7:**
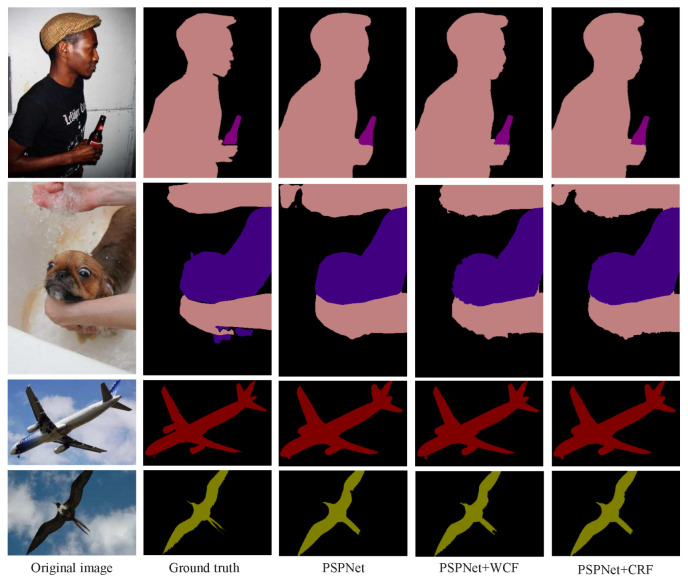
Enhancement of segmentation results of PSPNet. From the first row to the fourth row are man, dog, aircraft and bird, respectively. From the first column to the fifth column are the original images, the ground truth of images, the preliminary segmentation results by PSPNet, enhancement results by the proposed methods PSPNet+WCF and enhancement results by PSPNet+CRF.

The intuitive qualitative results of enhancement methods are presented in Figure 4, Figure 5, Figure 6 and Figure 7. In each figure, from the leftmost column to the rightmost are the original images, the ground truth of the image, the preliminary segmentation results and enhancement results. For the targets in the picture with many details, such as birds, airplanes, cats, dogs and persons, the preliminary segmentation results are difficult to display their contour edges. Furthermore, even only the main part can be simply presented because of the serious lack of edge information. The local characteristics of our method have a certain degree of trimming effect on the above-mentioned missing details, and can better display important details such as hair and facial contours. For the missing information in these preliminary segmentation results, such as the incomplete recognition of the lower body of a cat in the second row of Figure 5, and the wrong recognition of the target caused by misjudgment, such as misidentified hand in the second row of Figure 7, can also be complemented and corrected accordingly by the global characteristics of our methods.

**Figure 8:**
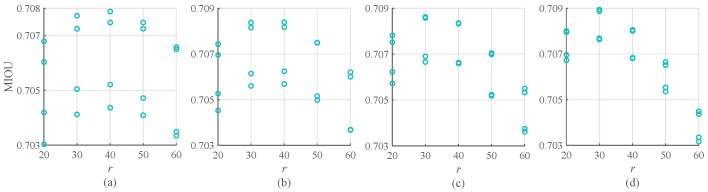
Parameters sensitivity demonstration of the proposed post-processing method combining PSPNet. (**a**) is the MIOU demonstration when $\omega_{1}=0.5$, σ changes from 5 to 20 with a step size of 5, and *r* changes from 20 to 60 with a step size of 5. (**b**) is the MIOU demonstration when $\omega_{1}=0.6$, σ changes from 5 to 20 with a step size of 5, and *r* changes from 20 to 60 with a step size of 5. (**c**) is the MIOU demonstration when $\omega_{1}=0.6$, σ changes from 5 to 20 with a step size of 5, and *r* changes from 20 to 60 with a step size of 5. (**d**) is the MIOU demonstration when $\omega_{1}=0.8$, σ changes from 5 to 20 with a step size of 5, and *r* changes from 20 to 60 with a step size of 5.

For quantitative evaluation, the criterions MIOU (Mean intersection over union) and PA (predicted segmentation) are used to show enhancement effects. As shown in Table 1, the proposed method is confirmed to indeed enhance the semantic segmentation results after being tested with 1499 images in the data set. It is worth mentioning that, as shown in Figure 8, when the parameters vary within a certain range, i.e., $\omega_{1}$ changes from 0.5 to 0.8 with a step size of 0.1, σ changes from 5 to 20 with a step size of 5, and *r* changes from 20 to 60 with a step size of 5, the corresponding MIOU changes only within a small range, which means that the parameter selection is more convenient and the workload of debugging of the learning process is reduced.

**Figure 9:**
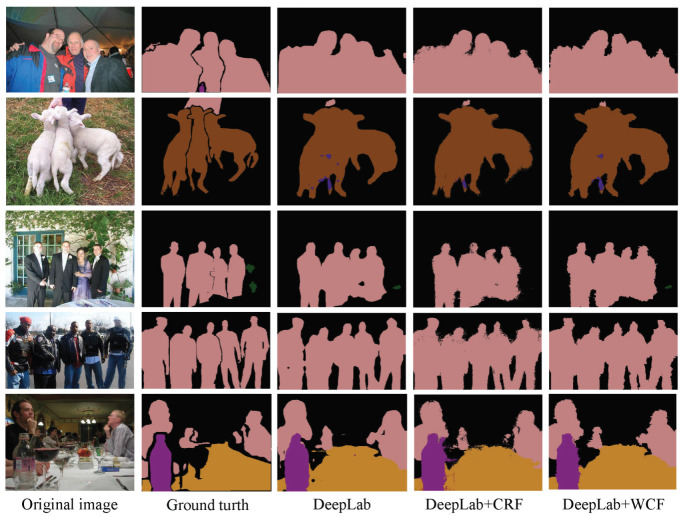
Enhancement of segmentation results of DeepLab for the complex scene with multiple objects of the same type. From the first column to the fifth column are the original images, the ground truth of images, the preliminary segmentation results by DeepLab, enhancement results by DeepLab+CRF and enhancement results by the proposed method DeepLab+WCF.

Meanwhile, the enhancement results of proposed method are compared with that of post-processing method based on CRF in [40]. As shown in Figure 7, the intuitive enhancement effects of the two post-processing methods are similar, but the wrong recognitions of the target caused by misjudgment are still not corrected in the CRF post-processing results. As shown in Table 2, the indicators MIOU and PA are higher than those of CRF and our method is superior to CRF. What’s more, the enhancement effects of the two post-processing techniques for the complex scene with multiple objects of the same type are shown in Figure 9. Although there is no specific training, both the methods can restore a certain degree of object details while ensuring integrity. The comparisons indicate the excellent application potential of our post-processing strategy.

## 5. Conclusions

In this paper, for the first time, we propose a novel post-processing method with theoretical simplicity in a unified framework to enhance the segmentation results and to solve the inherent problems in the current algorithm, such as poor detail identification, lack of spatial consistency and global information interaction, from the perspective of filtering. This allows us to have more choices to handle the enhancement issues when designing post-processing enhancement strategies besides the frameworks based on CRF. Meanwhile, a novel weight composite filter (WCF) that combines advantages of filters of both local type and global type is proposed by this method. The local characteristics of WCF ensure the clarity of boundaries and the completeness of details by utilizing local pixel coherence, and the global characteristics of WCF ensure the completeness and accuracy of segmentation targets by global information and spatial consistency. The effectiveness of our post-processing enhancement method and its range of application are validated through the experimental results. Besides, the comparison with the CRF-based strategy indicates the superiority of the proposed post-processing strategy. In the future, we will further improve the novel filters for posting processing and expand the application range of the proposed enhancement framework. In addition, the potential to generalize the proposed post-processing solution from the supervised algorithm to weakly supervised or unsupervised algorithms will also be investigated. 

## Figures and Tables

**Table 1:** Comparison of enhancement results of four semantic segmentation algorithms.

	Methods of Image Semantic Segmentation							
	FCN		FastFCN		DeepLab		PSPNet	
	FCN	FCN + WCF	FastFCN	FastFCN + WCF	DeepLab	DeepLab + WCF	PSPNet	PSPNet + WCF
MIOU	0.52448	**0.53490**	0.62857	**0.64315**	0.62937	**0.64314**	0.79395	**0.80239**
PA	0.88645	**0.89071**	0.90289	**0.90904**	0.90306	**0.90895**	0.95031	**0.95289**

**Table 2:** Comparison with enhancement results of CRF.

	Methods of Image Semantic Segmentation		
	PSPNet		
	PSPNet	PSPNet+CRF	PSPNet+WCF
MIOU	0.79395	0.79584	**0.80239**
PA	0.95031	0.95080	**0.95289**
